# Cervical screening in assertive outreach team patients

**DOI:** 10.1192/bjo.2021.299

**Published:** 2021-06-18

**Authors:** Hannah Reynolds, Samaila Bello, Hanna Leech

**Affiliations:** Birmingham and Solihull Mental Health NHS Foundation Trust

## Abstract

**Aims:**

To assess the uptake of cervical screening in patients under Birmingham and Solihull Assertive Outreach Teams; this included a re-audit of patients under the Central Birmingham Assertive Outreach Team.

**Background:**

Patients with severe and enduring mental illness are known to have poorer physical health outcomes. In Birmingham and Solihull there are 6 Assertive Outreach Teams. These teams manage patients with a diagnosis of psychosis who have complex needs requiring intensive multidisciplinary input and often struggle to engage with health services. The national cervical screening programme aims to prevent cervical cancer by detecting and treating cervical abnormalities. Acceptable coverage is defined as screening at least 80% of people aged 25–49 years within the last 3.5 years and 80% of people aged 50–64 years within the last 5.5 years. In 2018 71.4% of women in England and 70.9% in the West Midlands were screened adequately. An audit of 15 patients under the Central Birmingham Assertive Outreach Team in 2014 showed 46.2% had taken up screening, measured in the last 5 years for those aged 50–64 years and the last 3 years for those aged 25–49 years.

**Method:**

A list was obtained of all female patients under the Assertive Outreach Teams with patients excluded if they were under 25 years or over 64 years or if they were known to have undergone a total hysterectomy. All GP practices with eligible patients registered to them were written to requesting the date of the patient's most recent smear test. Cervical screening was classed as in date if carried out in the last 3.5 years for patients aged 25–49 years or 5.5 years for patients aged 50–64 years.

**Result:**

Out of 127 eligible patients, 110 had correct GP details on their record. Responses were received regarding 101 patients, 48 of whom had in date cervical screening (47.5%). Of 58 patients aged 25–49 years, 26 had in date cervical screening (44.8%). Of 43 patients aged 50–64 years, 22 had in date cervical screening (51.2%).

**Conclusion:**

13.4% patients did not have a known GP practice, increasing the risk of multiple poor physical health outcomes. The rates of cervical screening among Assertive Outreach Team patients are similar to the original audit in 2014 and fall significantly below the national standards and averages. These findings, along with the importance of working together to address the need for physical health monitoring in this population, will be communicated with the local Assertive Outreach Teams and GP practices.

